# Comparing prediction accuracy between total keratometry and conventional keratometry in cataract surgery with refractive multifocal intraocular lens implantation

**DOI:** 10.1038/s41598-021-98491-x

**Published:** 2021-09-28

**Authors:** Ho Seok Chung, Jae Lim Chung, Young Jun Kim, Hun Lee, Jae Yong Kim, Hungwon Tchah

**Affiliations:** 1 Department of Ophthalmology, Dankook University Hospital, Dankook University College of Medicine, Cheonan, South Korea; 2 Eyejun Ophthalmic Clinic, Seoul, South Korea; 3 Department of Ophthalmology, College of Medicine, University of Ulsan, Asan Medical Center, 88, Olympic-ro 43-gil, Songpa-gu, Seoul, 05505 South Korea

**Keywords:** Lens diseases, Refractive errors

## Abstract

We aimed to compare refractive outcomes between total keratometry using a swept-source optical biometer and conventional keratometry in cataract surgery with refractive multifocal intraocular lens (IOL) implantation. We included patients who underwent cataract surgery with refractive multifocal IOL implantation. The IOL power was calculated using conventional formulas (Haigis, SRK/T, Holladay 2, and Barrett Universal II) as well as a new formula (Barrett TK Universal II). The refractive mean error, mean absolute error, and median absolute error were compared, as were the proportions of eyes within ± 0.25 diopters (D), ± 0.50 D, and ± 1.00 D of prediction error. In total 543 eyes of 543 patients, the absolute prediction error of total keratometry was significantly higher than that of conventional keratometry using the SRK/T (*P* = 0.034) and Barrett Universal II (*P* = 0.003). The proportion of eyes within ± 0.50 D of the prediction error using the SRK/T and Barrett Universal II was also significantly higher when using conventional keratometry than total keratometry (*P* = 0.010 for SRK/T and *P* = 0.005 for Barrett Universal II). Prediction accuracy of conventional keratometry was higher than that of total keratometry in cataract surgery with refractive multifocal IOL implantation.

## Introduction

When estimating total keratometry (TK), clinicians must consider posterior as well as anterior keratometry to ensure they obtain accurate measurements. Traditionally, anterior corneal measurements have been used to estimate posterior corneal power based on a fixed posterior:anterior curvature ratio. Both TK and astigmatism can then be estimated based on anterior corneal measurements combined with an estimated posterior corneal power. In one published study, the measured TK showed a strong correlation with the TK estimated using a Goggin nomogram, which is based on the anterior keratometric value^1^.

The IOLMaster 700 (Carl Zeiss Meditec, Jena, Germany), which is based on swept-source optical coherence tomography, was developed to allow more precise optical biometry. It can measure the correct axial length (AXL) by imaging a cross section of the eye. The IOLMaster 700 also can measure the TK and calculate the exact intraocular lens (IOL) power using exclusive formulas, such as the Barrett TK Universal II and the Barrett TK toric^2^. Several articles have applied the currently used IOL calculation formulas to both the measured TK and conventional keratometry (K) to calculate the refractive outcomes of cataract surgery using monofocal or toric IOLs, while others have described cataract surgery performed after refractive surgery^2–6^.

Cataract surgery technology is constantly evolving, with increasing predictive accuracy and more sophisticated refractive results. In cataract surgery with multifocal IOL implantation, which has recently become more common, accurate IOL power is important for postoperative distant and near visual quality and is determined using optical biometric measurements.

Our group recently reported differences between prediction errors in applying K and TK data according to the type of diffractive multifocal IOL^7^. According to another recently published paper, the application of TK to formulas used after refractive surgery was not beneficial in cataract surgery after refractive surgery^8^. As such, it remains unclear whether TK in cataract surgery with refractive multifocal IOL implantation is useful, and long-term data are needed. In the present study, we compared the prediction accuracy of TK with that of conventional K in cataract surgery with refractive multifocal IOL implantation.

## Results

**Figure 1:**
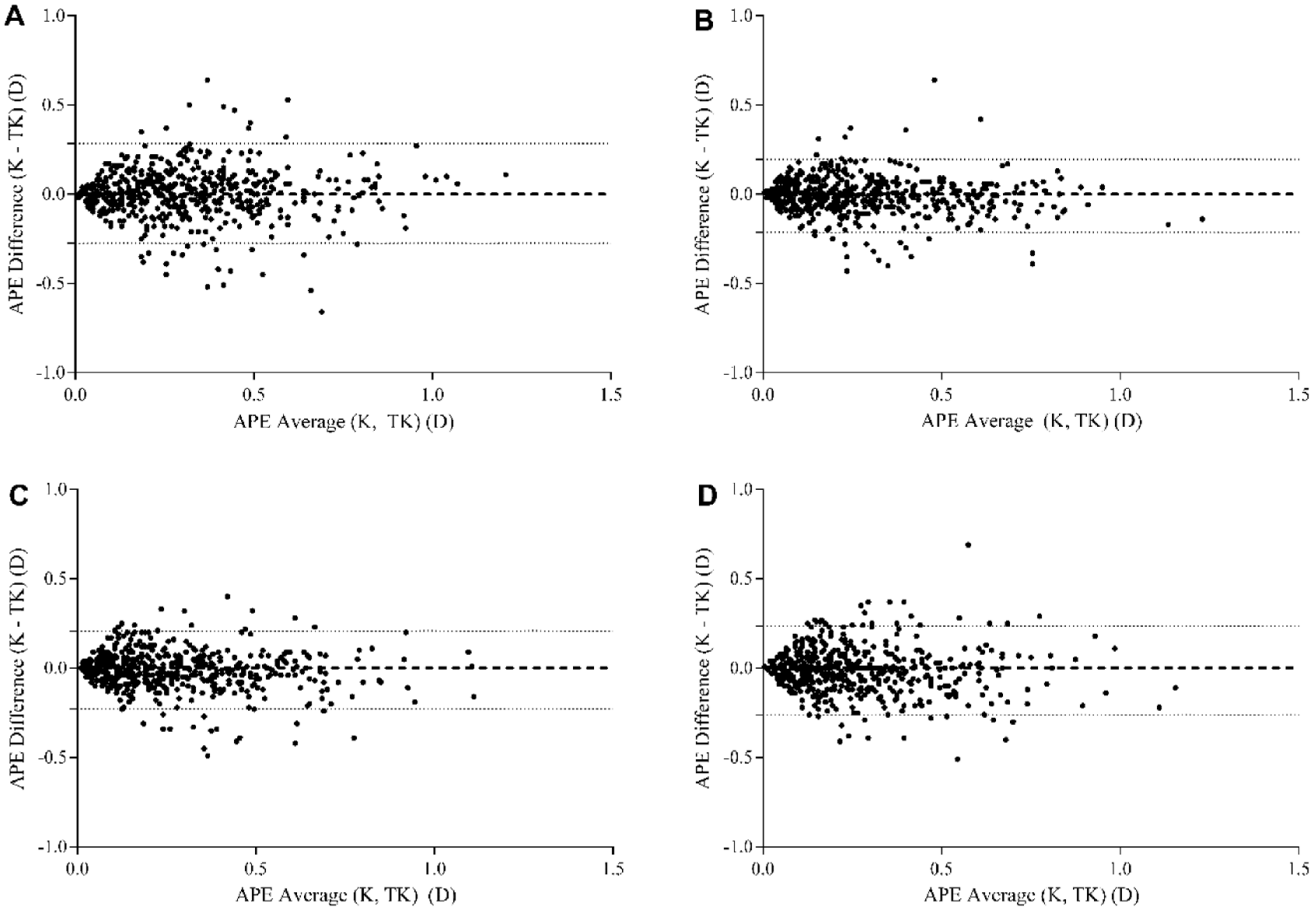
Bland–Altman plots comparing absolute predictive error between conventional keratometry and total keratometry using each formula (**A**) Haigis; (**B**) SRK/T; (**C**) Holladay 2; (**D**) Barrett Universal II. Ranges within 95% of values are indicated by dot lines. *APE* absolute predictive error, *K* conventional keratometry, *TK* total keratometry.

**Figure 2:**
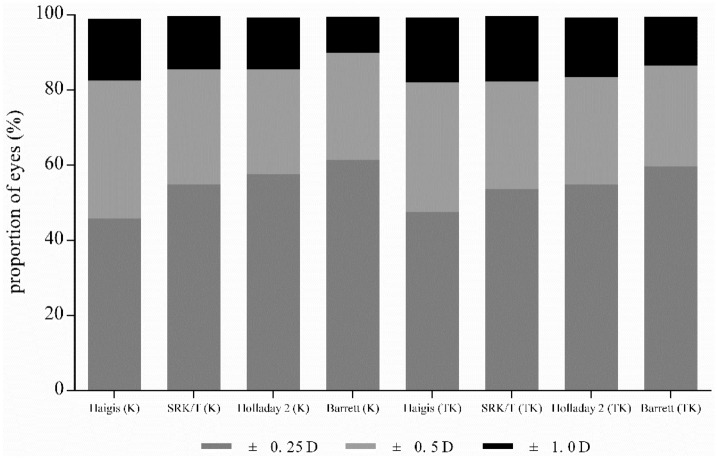
Proportional graph of eyes within ± 0.25 D, ± 0.50 D, and ± 1.00 D of prediction error of each formula using conventional keratometry and total keratometry. *K* conventional keratometry, *TK* total keratometry.

A total 543 eyes of 543 patients were included in the present study. The preoperative demographics of the patients and number of patients in each AXL subgroup are shown in Table 1. Table 2 shows the refractive mean error (ME), mean absolute error (MAE), and median absolute error (MedAE) according to each formula using either K or TK. When comparing the absolute predictive error (APE) using the K and TK calculated by each formula, the SRK/T and Barrett Universal II formulas showed significantly lower APE with K compared to TK (*P* = 0.127 for Haigis, *P* = 0.034 for SRK/T, *P* = 0.097 for Holladay 2, and *P* = 0.003 for Barrett Universal II). Figure 1 shows the Bland–Altman plot of the APE differences between K and TK calculated using each formula. The proportions of eyes within ± 0.25 diopters (D), ± 0.50 D, and ± 1.00 D of the prediction error using K and TK according to each formula are shown in Table 3 and Fig. 2. When comparing the proportion of eyes within ± 0.50 D between K and TK, SRK/T (*P* = 0.010) and Barrett Universal II (*P* = 0.005) formulas showed a significantly higher proportion of K compared to TK.

**Table 1:** Patients’ demographics and biometric measurements.

Variable	
Age (years)	58.26 ± 5.71 (43–74)
Male/female	132/411
Right/left	267/276
Preoperative BCVA (logMAR)	0.063 ± 0.06
**Axial length (mm)**	23.55 ± 1.02 (20.85–28.38)
Short (< 22.5 mm)	60 (11.0%)
Medium (22.5–25.5 mm)	462 (85.1%)
Long (> 25.5 mm)	21 (3.87%)
Anterior chamber depth (mm)	3.13 ± 0.32 (2.26–4.04)
Mean conventional keratometry (D)	44.21 ± 1.29 (39.75–47.88)
IOL power (D)	20.14 ± 2.79 (8.50–27.00)

*BCVA* best corrected visual acuity, *D* diopters, *IOL* intraocular lens.

**Table 2:** Refractive mean error, mean absolute error, and median absolute error, according to each formula, using conventional keratometry or total keratometry.

	ME	SD	MAE	MedAE
Haigis (K)	0.196	0.335	0.317	0.270
Haigis (TK)	0.167	0.345	0.312	0.270
SRK/T (K)	− 0.046	0.343	0.275	0.230
SRK/T (TK)	− 0.073	0.353	0.284	0.240
Holladay 2 (K)	− 0.039	0.337	0.268	0.210
Holladay 2 (TK)	− 0.077	0.347	0.278	0.230
Barrett (K)	− 0.054	0.315	0.251	0.210
Barrett (TK)	− 0.107	0.318	0.264	0.210

*ME* mean error, *SD* standard deviation, *MAE* mean absolute error, *MedAE* median absolute error, *K* conventional keratometry, *TK* total keratometry.

**Table 3:** Proportion of eyes within ± 0.25 D, ± 0.50 D, and ± 1.00 D of spherical equivalent prediction error.

IOL formula	± 0.25 D		± 0.50 D		± 1.00 D	
	K (%)	TK (%)	K (%)	TK (%)	K (%)	TK (%)
Haigis	45.9	47.5	82.5	82.1	98.9	99.3
SRK/T	54.9	53.6	85.5*	82.3*	99.6	99.6
Holladay 2	57.6	54.9	85.5	83.4	99.3	99.3
Barrett	61.3	59.7	89.9*	86.6*	99.4	99.4

*D* diopters, *K* conventional keratometry, *TK* total keratometry.

*Statistically significant difference between the K and TK.

**Figure 3:**
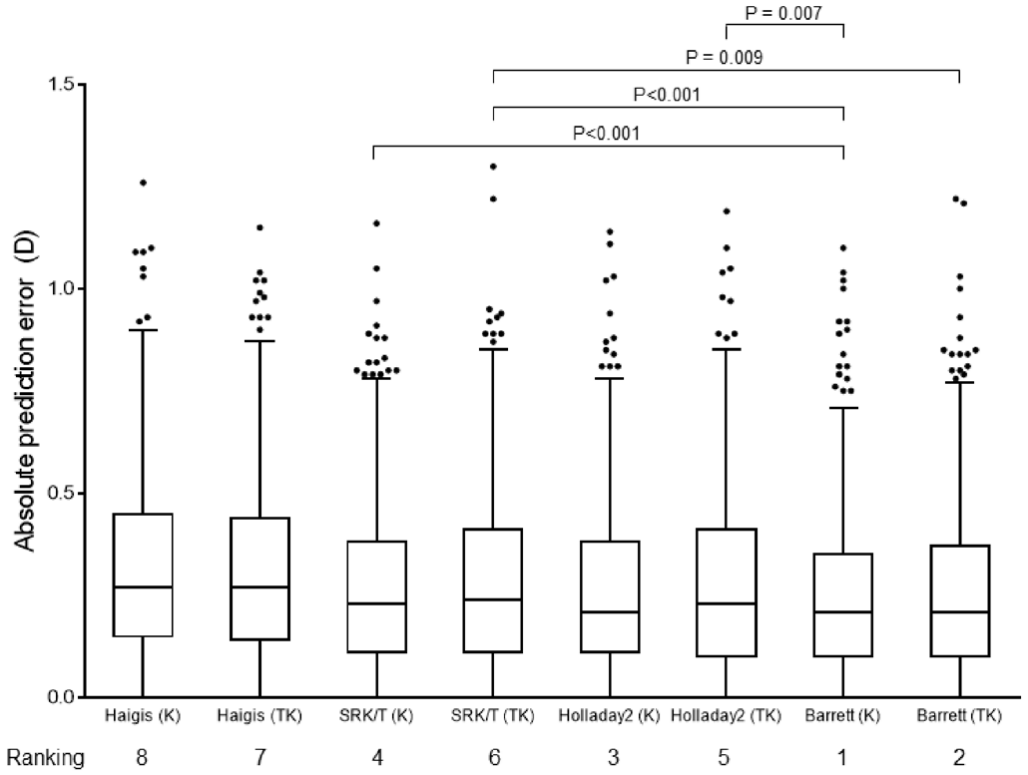
Box and whisker plots and rankings from lowest APE when applying the Haigis, SRK/T, Holladay 2, and Barrett Universal II/Barrett TK Universal II formulas using K and TK. P-values adjusted for use of multiple comparisons are indicated in this figure. *K* conventional keratometry, *TK* total keratometry.

There was a significant difference in APE among all the formulas (P < 0.001). Post hoc analysis was conducted using the Wilcoxon signed-rank test, with a Bonferroni correction applied, resulting in a significance level of P < 0.0018. The overall comparison and post hoc analysis showed that the APE was lowest in the Barrett Universal II using K, followed in order by the Barrett TK Universal II, the Holladay 2 using K, the SRK/T using K, the Holladay 2 using TK, the SRK/T using TK, the Haigis using TK, and the Haigis using K (Fig. 3). When each formula was compared with all others, the APE of the Haigis using K was significantly higher from that of all other formulas, and the APE of the Haigis using TK was significantly higher from that of all formulas except for SRK/T using TK. Among the other formulas, there were significant differences between SRK/T using K and Barrett Universal II using K (adjusted P < 0.001), between SRK/T using TK and Barrett Universal II using K (adjusted P < 0.001), between SRK/T using TK and Barrett TK Universal II (adjusted P = 0.009), and between Holladay 2 using TK and Barrett Universal II using K (adjusted P = 0.007).

Table 4 shows the AXL subgroup analysis. In short eyes, the Barrett TK Universal II had the lowest MAE, while the Haigis using K had the highest MAE. In long eyes, the Barrett Universal II using K had the lowest MAE, while the Haigis using TK had the highest MAE.

**Table 4:** Mean absolute error in each axial length subgroup, according to the formulas.

	Short AXL	Medium AXL	Long AXL
Haigis (K)	0.345	0.311	0.330
Haigis (TK)	0.338	0.306	0.333
SRK/T (K)	0.337	0.266	0.265
SRK/T (TK)	0.318	0.278	0.276
Holladay 2 (K)	0.334	0.257	0.279
Holladay 2 (TK)	0.324	0.270	0.281
Barrett (K)	0.304	0.243	0.243
Barrett (TK)	0.282	0.258	0.309

Short eyes (< 22.5 mm); medium eyes (22.5–25.5 mm); long eyes (> 25.5 mm).

*K* conventional keratometry, *TK* total keratometry, *AXL* axial length.

## Discussion

In the present study, we demonstrated that the prediction accuracy of conventional K was higher than that of TK in cataract surgery with refractive multifocal IOL implantation. When using the SRK/T and Barrett Universal II formulas, K had a significantly higher prediction accuracy than TK. Previous studies on the clinical usefulness of TK have used several kinds of monofocal or toric IOLs. In the present study, we focused on the use of refractive multifocal IOLs to investigate the accuracy of TK in IOL power prediction. In addition, one strength of the study was its large sample size.

With all formulas except the Haigis formula, MAE and MedAE were higher when using TK than when using K, and the difference was significant in the SRK/T and Barrett Universal II formulas. When comparing the proportion of eyes within ± 0.50 D of the prediction error, K showed a significantly higher proportion than TK in the SRK/T and Barrett Universal II formulas. Previous studies have reported that, when the Barrett Universal II and SRK/T formulas were applied to conventional K, the MAE and MedAE were higher than when those formulas were applied to TK^2,3^. The present study contradicts this finding. It may be that the SRK/T formula showed more accurate results when using conventional K because it was developed using that measurement. However, the Barrett TK Universal II was developed based on TK, so by the same reasoning one would expect more accurate results when TK was applied to Barrett TK Universal II than when K was applied to Barrett Universal II.

These unexpected results may have occurred because the two previous studies used the 601P/PY IOL (Carl Zeiss Meditec, Jena, Germany) and Asphina 409M/MP IOL (Carl Zeiss Meditec, Jena, Germany), respectively, whereas the present study used multifocal IOLs from different manufacturer. In addition, when comparing the proportion of eyes within ± 0.50 D of the prediction error, Fabian et al. reported 77% and 84%, respectively, when applying K to the Haigis and Barrett Universal II formulas, and 79% and 86% when applying TK to the same formulas^2^. Sirvannaboon et al. reported 62% and 60%, respectively, when applying K to the Haigis and Barrett Universal II formulas, and 63% and 67% when applying TK to them^3^. In the present study, we found values of 82.5% and 89.9%, respectively when applying K to the Haigis and Barrett Universal II formulas, and 82.1% and 86.6% when the TK was applied. Compared to the two studies mentioned, when using TK, the prediction accuracy was similar or higher, while when using K, the accuracy was much higher. Therefore, the unexpected results of the present study likely resulted from higher accuracy when using K rather than from low accuracy when using TK. The patients included in the present study underwent multifocal IOL implantation to correct presbyopia. As such, they were younger and better preoperative best corrected visual acuity (BCVA) than the patients in other IOL power calculation studies. Therefore, it may be that we could make more accurate biometric measurements and that the IOL power calculation had greater accuracy.

The Haigis formula was the only one that showed lower MAE when using TK than when using K. In a previous study, Haigis using TK exhibited higher accuracy than Haigis using K^2^. When estimating the cumulative percentage of eyes, Haigis K showed 77% within 0.50 D and 95% within 1.00 D, while Haigis TK showed 79% within 0.50 D and 96% within 1.00 D. All of these proportions were higher in the present study than in the previous study^2^.

When comparing the APE between formulas, not all comparisons were significant, but the overall comparison was significant (Fig. 3). In a recently published study on TK, when using both K and TK, MAE and MedAE was lowest when applying Barrett Universal II, followed by SRK/T, Holladay 2, and Haigis^3^. Our study found similar results, although the order between SRK/T and Holladay 2 was reversed, and the difference was not significant using either K or TK. As mentioned above, the Barrett TK Universal II formula showed significantly inferior results than the Barrett Universal II using K, but it did show superior results compared to other formulas using K, perhaps because the Barrett TK Universal II was developed based on TK. Conversely, when the previously used formulas SRK/T, Holladay 2, and Haigis used TK, the result was inferior to all formulas using K except Haigis.

A review article on IOL power calculation mentioned that the Haigis and Holladay 2 formulas have higher accuracy than the Barrett Universal II formula in short eyes, and that the Barrett Universal II, Haigis, Olsen, and SRK/T formulas have greater accuracy in long eyes^9^. In the AXL subgroup analyses of the present study, Barrett formulas resulted in the lowest MAE, regardless of the AXL—Barrett TK Universal II in short eyes, Barrett Universal II in medium and long eyes, while Haigis formulas conferred the highest MAE—Haigis K in short and medium eyes, Haigis TK in long eyes. Both Haigis and Holladay 2, which were mentioned as high accuracy in the review article, showed high MAE in short eyes, and Haigis even showed high MAE in long eyes, whereas Barrett and SRK/T showed lower MAE in long eyes than in short eyes, showing high accuracy in long eyes. Although these results were somewhat contrary to those previously published, our results included only 11.0% of all subjects for short eyes and 3.87% for long eyes, so a larger number of patients should be analysed in a future study.

The present study was limited by its retrospective nature and because the average ME was not set to zero using the A constant as a User Group of the Laser Interference Biometry (ULIB). To allow more accurate IOL power prediction, an optimally personalized lens constant that can set the average ME to 0 should be applied to eliminate bias caused by the lens factor. Excluding this limitation, the present study followed all protocols suggested for studies into IOL formula accuracy proposed by Hoffer et al.^10^. The cataract surgery in the present study was performed by one expert surgeon using only one IOL model, one eye from each patient was randomly included, and postoperative refraction was measured 2 months after surgery.

In conclusion, when applying the formulas currently in use to TK during cataract surgery with refractive multifocal IOL implantation, the refractive results were not superior to those of K. The APE was higher when using TK than when using K, so more TK data must be gathered for each formula to allow more accurate IOL power calculation using TK.

## Methods

### Subject

This study was conducted with the approval of the Institutional Review Board of the Asan Medical Center and University of Ulsan College of Medicine, Seoul, South Korea (Approval number: 2020-1290). The study adhered to the tenets of the Declaration of Helsinki and followed good clinical practice guidelines. This study was conducted retrospectively and the informed consent from the patients was waived.

In this retrospective study, all patients underwent cataract surgery with phacoemulsification and implantation of refractive multifocal IOL (Lentis M plus; Oculentis GmbH., Berlin, Germany). All procedures were performed by a single experienced surgeon. In previous studies, this refractive multifocal IOL has shown stable distant and near vision, as well as fewer photopic phenomena and better intermediate vision than diffractive multifocal IOLs^11,12^. The study included patients who underwent cataract surgery between January 2019 and December 2019, who underwent a complete preoperative examination, uncomplicated cataract surgery, and a follow-up of at least 2 months. Patients who had undergone previous intraocular surgery were excluded, as were those with a BCVA of less than 0.1 logMAR at 2 months after surgery. One eye was randomly selected to participate in the study. All subjects underwent a complete ophthalmologic examination before surgery, including BCVA and automated keratometry using the RK-F2 Full Auto Ref-Keratometer (Canon, Tokyo, Japan). The AXL, anterior chamber depth, K, and TK were measured using the IOLMaster 700. At least 2 months after surgery, patients underwent ophthalmologic examination, including BCVA, automated keratometry, and manifest refraction. The parameters were measured twice by an experienced technician to ensure accuracy. Patients were categorised into three groups according to the preoperative axial length: short (< 22.5 mm), medium (22.5–25.5 mm), and long (> 25.5 mm).

### Calculating IOL prediction error

IOL power calculations were carried out on an IOLMaster 700 unit (Barrett Suite, software version 1.80.6.60340). We applied the TK and K of all patients to the currently widely used formulas (Haigis, SRK/T, Holladay 2). In the Barrett formula, K is applied to Barrett Universal II, and TK is applied to Barrett TK Universal II, a new formula based on TK. We used the lens constants of the User Group of the Laser Interference Biometry (ULIB) website.

ME was defined as the mean value obtained by subtracting the preoperative predicted spherical equivalent from the actual spherical equivalent. APE was defined as the absolute value obtained by subtracting the actual spherical equivalent from the preoperative predicted spherical equivalent. MAE was defined as the mean APE value, while MedAE was defined as the median APE. The proportion of eyes within ± 0.25 D, ± 0.50 D, and ± 1.00 D of the prediction error were measured.

### Statistical analyses

A Wilk–Shapiro test was used to assess the distribution of numerical data. The Wilcoxon signed-rank test was used to compare the APE between K and TK. The APE was compared between the formulas using the Friedman test, with Bonferroni post-hoc correction for multiple comparisons. The proportions of eyes within ± 0.25 D, ± 0.50 D, and ± 1.00 D were compared using the McNemar’s test. For each formula, APE differences between conventional K and TK were obtained, the differences were analysed using the Bland–Altman plot. All data were statistically analysed using SPSS software version 25.0 (IBM, Armonk, NY, USA), and all P-values less than 0.05 were considered statistically significant.

## Data Availability

The datasets generated and analysed during the current study are available from the corresponding author on reasonable request.
